# Accuracy of Prospective Assessments of 4 Large Language Model Chatbot Responses to Patient Questions About Emergency Care: Experimental Comparative Study

**DOI:** 10.2196/60291

**Published:** 2024-11-04

**Authors:** Jonathan Yi-Shin Yau, Soheil Saadat, Edmund Hsu, Linda Suk-Ling Murphy, Jennifer S Roh, Jeffrey Suchard, Antonio Tapia, Warren Wiechmann, Mark I Langdorf

**Affiliations:** 1 College of Natural and Agricultural Sciences University of California - Riverside Riverside, CA United States; 2 Department of Emergency Medicine University of California - Irvine Orange, CA United States; 3 Reference Department University of California - Irvine Libraries Irvine, CA United States; 4 Department of Emergency Medicine Harbor-UCLA Medical Center University of California - Los Angeles Torrance, CA United States

**Keywords:** artificial intelligence, AI, chatbots, generative AI, natural language processing, consumer health information, patient education, literacy, emergency care information, chatbot, misinformation, health care, medical consultation

## Abstract

**Background:**

Recent surveys indicate that 48% of consumers actively use generative artificial intelligence (AI) for health-related inquiries. Despite widespread adoption and the potential to improve health care access, scant research examines the performance of AI chatbot responses regarding emergency care advice.

**Objective:**

We assessed the quality of AI chatbot responses to common emergency care questions. We sought to determine qualitative differences in responses from 4 free-access AI chatbots, for 10 different serious and benign emergency conditions.

**Methods:**

We created 10 emergency care questions that we fed into the free-access versions of ChatGPT 3.5 (OpenAI), Google Bard, Bing AI Chat (Microsoft), and Claude AI (Anthropic) on November 26, 2023. Each response was graded by 5 board-certified emergency medicine (EM) faculty for 8 domains of percentage accuracy, presence of dangerous information, factual accuracy, clarity, completeness, understandability, source reliability, and source relevancy. We determined the correct, complete response to the 10 questions from reputable and scholarly emergency medical references. These were compiled by an EM resident physician. For the readability of the chatbot responses, we used the Flesch-Kincaid Grade Level of each response from readability statistics embedded in Microsoft Word. Differences between chatbots were determined by the chi-square test.

**Results:**

Each of the 4 chatbots’ responses to the 10 clinical questions were scored across 8 domains by 5 EM faculty, for 400 assessments for each chatbot. Together, the 4 chatbots had the best performance in clarity and understandability (both 85%), intermediate performance in accuracy and completeness (both 50%), and poor performance (10%) for source relevance and reliability (mostly unreported). Chatbots contained dangerous information in 5% to 35% of responses, with no statistical difference between chatbots on this metric (*P*=.24). ChatGPT, Google Bard, and Claud AI had similar performances across 6 out of 8 domains. Only Bing AI performed better with more identified or relevant sources (40%; the others had 0%-10%). Flesch-Kincaid Reading level was 7.7-8.9 grade for all chatbots, except ChatGPT at 10.8, which were all too advanced for average emergency patients. Responses included both dangerous (eg, starting cardiopulmonary resuscitation with no pulse check) and generally inappropriate advice (eg, loosening the collar to improve breathing without evidence of airway compromise).

**Conclusions:**

AI chatbots, though ubiquitous, have significant deficiencies in EM patient advice, despite relatively consistent performance. Information for when to seek urgent or emergent care is frequently incomplete and inaccurate, and patients may be unaware of misinformation. Sources are not generally provided. Patients who use AI to guide health care decisions assume potential risks. AI chatbots for health should be subject to further research, refinement, and regulation. We strongly recommend proper medical consultation to prevent potential adverse outcomes.

## Introduction

There has been a significant surge in attention to artificial intelligence (AI)–driven chatbots capable of generating content and engaging in conversations resembling natural human communication. While using a smartphone or a computer, most people have interacted with a chatbot—software or computer program that simulates human conversation [1,2]. Modern chatbots use generative AI (GenAI) technologies such as machine learning, large language models (LLMs), natural language processing, and deep learning to more accurately interpret user questions and interact in a natural way. Furthermore, GenAI recognizes, predicts, and can create content without human interaction [3,4].

Since their release to the general public, chatbots such as ChatGPT, Google Bard (now known as Gemini) [5], Bing AI Chat (now known as Copilot) [6], and Claud AI have penetrated diverse industries, including technology, education, research, health care, finance, and social media. In health care, LLMs have demonstrated the potential to revolutionize the field, with ChatGPT able to achieve a passing score on the US Medical Licensing Examination and write basic medical reports [7-9]. Future applications of LLMs are vast, including assisting with medical education, predicting diagnoses, and recommending treatment options to patients [10].

Consumers have turned to the web as their first source of information for many medical-related inquiries. The National Center for Health Statistics (NCHS) data brief published by the National Center for Health Statistics found that 58.5% of US adults used the internet to look for health or medical information in 2022. The main motivations were convenience and breadth of information, which were less available through traditional doctor visits and access to interpretation of medical test results [11]. A more recent 2024 survey by Deloitte found that nearly half (48%) of consumer respondents have used GenAI for health-related concerns, citing its potential to make health care more accessible, affordable, and reliable [12]. Finally, in 2023, a survey reported that 78.4% of subjects would be willing to use Chat-GPT for self-diagnosis [13]. This growing reliance on GenAI highlights the importance of further research on whether current LLMs provide accurate, relevant answers to medical questions across specialties.

Access to medical information is a social determinant of health [14,15]. GenAI, therefore, has the potential to both mitigate or exacerbate health disparities, depending on its accuracy and completeness. While younger patients who are facile with English and technology may be better able to access web-based health information, older patients or those whose primary language is not English are more at risk for reduced access or response comprehension. Furthermore, patients with more limited access to physicians due to insurance barriers could be more apt to use GenAI chatbots for medical queries, adding to their disadvantages in accessing proper health care.

Since the release of ChatGPT 3.5 on November 30, 2022, there has been a substantial increase in medical literature related GenAI in health care. Nearly 4000 publications were found in PubMed between November 30, 2022, and May 1, 2024. A 2023 study analyzing ChatGPT responses to questions across 17 different specialties found ChatGPT to be extremely promising, with the chatbot scoring high in median accuracy and completeness. However, the researchers noted that the chatbot would sometimes be “spectacularly and surprisingly wrong” [16]. Another study examined ChatGPT’s ability to answer bariatric surgery-related questions and found the responses to be mostly comprehensive and reproducible [17]. Similar studies seem to report that ChatGPT produces largely accurate information. However, ChatGPT’s inability to cite up-to-date, reputable sources in its responses may result in sporadic-to-frequent occurrences of blatant misinformation [18].

This paper expands the literature on chatbot reliability in 3 ways. First, we used board-certified emergency medicine (EM) faculty physicians as assessors of the accuracy, safety, completeness, readability, and reliability of emergency care information. Second, we asked questions of the chatbots about common potentially emergent conditions, which, if inaccurate, could exacerbate negative social determinants of health. Wrong or missing information could be dangerous. Third, we judged the responses against the reading or language capabilities of average emergency patients. Studies suggest most emergency patients read below or at the eighth-grade level [19,20]. The American Medical Association (AMA) recommends a maximum fifth-grade reading level for health literacy materials directed toward Medicaid enrollees [21].

Additionally, we assessed the performance of less commonly used consumer chatbots, such as Google Bard, Bing AI, and Claude AI, which have not been as rigorously assessed as those of ChatGPT.

Our objective was to comprehensively evaluate and compare the strengths and weaknesses of answers from ChatGPT, Google Bard, Bing AI, and Claude AI to common emergency care questions, as judged by board-certified EM faculty physicians, who provide high-level expertise and clinical relevance in the evaluation process.

## Methods

### Overview

The research prompts were 10 common emergency care questions by patients, selected by 5 board-certified EM faculty (EH, MIL, JSR, JS, and WW) from common emergency department (ED) chief complaints. These were representative of both benign and potentially serious conditions [22]. The 10 question topics were chest pain, stroke, bad headache, bad sore throat, bad stomach pain, bad back pain, fainting, heavy menstrual bleeding, bad cold, and drug overdose.

We included these based on a reference [22] that described the most common chief complaints for EDs. These included abdominal pain, shortness of breath, chest pain, and 4 neurological complaints, which we included with our choice of back pain, stroke, fainting or syncope, and headache. Other chief complaints we chose to test (heavy period bleeding and sore throat) were found in a lower proportion of patients and are listed in a footnote. “Common cold” is not specifically listed as a common chief complaint in this reference, inexplicably, but could be subsumed in shortness of breath and dysphagia or sore throat.

The wording of each question was then refined to reflect the language typically used by patients, with emphasis on what actions the inquirer should take. The finalized prompts are listed in Multimedia Appendix 1.

To minimize bias, the chatbots were accessed through a newly created email account on a browser with cleared cookies and cache. For each prompt, a new chat was initiated, and the chatbot’s first response or draft was documented (Multimedia Appendix 2). If the chatbot did not list sources in the initial response, a follow-up query was entered—“Please list all sources of information you referenced.” All responses were generated on November 26 and 27, 2023, using publicly available free versions of the chatbots at that time. We chose free versions, as, at the moment of a patient wondering what to do about a clinical problem, they would likely query the free version of a chatbot, rather than sign up for access to a more sophisticated, fee-based version.

As a benchmark for grading the chatbot-generated responses, “correct” answers to the 10 questions were developed (Multimedia Appendix 3) by an emergency medical resident (AT) citing trusted medical sources—“Patient Education - UpToDate,” MedlinePlus Health Topics, and Mayo Clinic (full references listed in Appendix 3). A total of 5 board-certified emergency faculty (EH, MIL, JSR, JS, and WW) validated the completeness and accuracy of each response to these 10 questions from the 4 chatbots.

We assessed the reliability of chatbots for emergency medical advice with a standardized grading rubric to mitigate evaluator bias and enhance objectivity. While the study by Altamimi et al [23] evaluated ChatGPT by clinical toxicologists and emergency physicians for providing recommendations about venomous snakebites, the lack of a defined grading scale raised concern regarding potential bias in their evaluation process [23].

Given the absence of an accepted standard to evaluate chatbot-generated medical advice [24-29], we developed our own comprehensive scoring sheet. Drawing inspiration from the Academic Life in Emergency Medicine Approved Instructional Resources rating score and evaluative scales from prior chatbot studies [17,30-34], our scoring sheet facilitates straightforward documentation of evaluations via a Google Form. It rigorously assesses each response based on 8 criteria, which we called “domains”—accuracy, safety, factual accuracy, clarity, completeness, readability, source availability, and source reliability (Multimedia Appendix 4). We defined a qualitative and quantitative measure of accuracy. The first was a percentage of correct information by quartile, dubbed “accuracy,” and the second, “factually accurate,” was whether the responses contained none, 1, 2, or more than 2 minor, major, or minor inaccuracies.

EM faculty identified significant omissions from the chatbot responses as 3 types. The first type was frank omissions of specific required advice, such as no mention of naloxone for opioid overdoses, no mention that antibiotics do not help most sore throats or common colds, and no mention of aspirin for chest pain. The second type was global missing concepts, such as what specifically should prompt medical attention (so-called “red flag” symptoms) and how long one should wait before deciding to seek medical attention. The third type was complete neglect of life-threatening diagnoses that should be considered with the presenting complaint (eg, no mention of pregnancy, either ectopic or intrauterine, for heavy menstrual bleeding). Some of these incomplete elements were judged to overlap with reported dangerous conditions.

We calculated the Flesch-Kincaid Grade Level (FKGL) of each of the 40 chatbot responses using the embedded FKGL tool within Microsoft Word for Microsoft 365 MSO (version 2402 build 16.0.17328.20124).

Reviewers’ ratings, on a 5-point ordinal scale, were dichotomized into “highest/best score” (ie, score 5) versus “less than perfect score” (ie, scores 1-4). The relative frequency of the “highest/best score” category was calculated and compared across the 4 chatbots, 10 medical conditions, and 8 domains of interest. The chi-square test was used to compare the relative frequency of “highest/best score” among the variables of interest (chatbots, medical conditions, and domains). For each comparison, *P* values were estimated using the asymptotic 2-way approximation. When the assumptions of asymptotic approximation were not met, the Fisher exact test or Monte Carlo simulations with 10,000 replications were used to obtain a more accurate *P* value. We report 99% CIs for the estimated *P* values. For the FKGL reading level score, the average and corresponding 95% Cl were calculated and presented as an error bar chart. EM faculty raters’ agreement was examined by using the Gwet Agreement Coefficient 1 (AC1) statistic due to its applicability to data with ordinal scales. The Gwet AC1 statistic was estimated by the *kappaetc* package [35], on Stata (version 17; StataCorp).

### Ethical Considerations

This project did not involve any human participants as actual patients who queried the chatbots. The US Food and Drug Administration (FDA) regulations define a human participant as “an individual who is or becomes a participant in research, either as a recipient of the test article or as a control.” Since the role of the EM faculty in our study was to adjudicate the accuracy of the chatbot responses, we did not find it necessary to seek an ethics review board assessment based on the guidelines set by the University of California, Irvine institutional review board [36].

## Results

### Overview

Five reviewers scored ChatGPT, Claude AI, Bing AI, and Google Bard for the medical conditions of chest pain, stroke, headache, sore throat, stomach pain, back pain, fainting, heavy menstrual bleeding, bad cold, and overdose. The overall agreement between the raters for all chatbots and across all clinical queries was moderate (Gwet AC1: 0.486; 0.463-0.508; scale 0-1 with 1 denoting perfect agreement). Gwet AC1 ranged between 0.445 and 0.574 among the scored domains.

**Figure 1:**
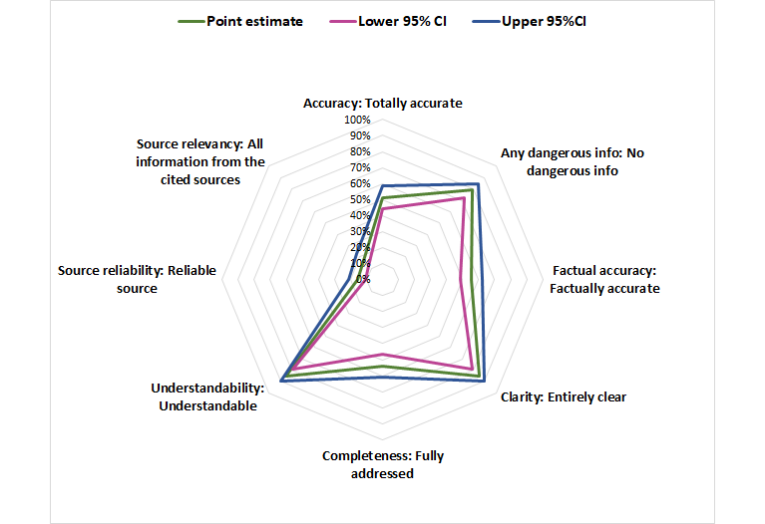
Performance of all 4 chatbots in aggregate across 8 different domains, showing the point estimate of the prevalence of the highest or best score in that domain, with the 95% CI.

**Figure 2:**
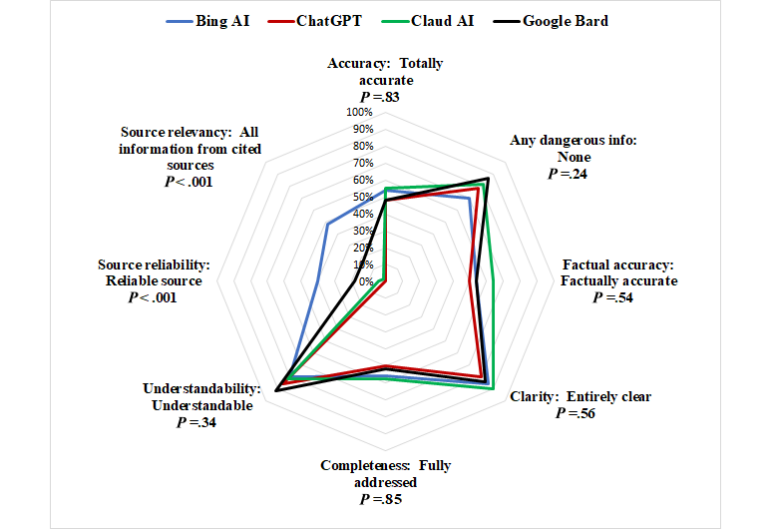
Comparison of the 4 chatbots’ performance against each other in the 8 domains.

**Figure 3:**
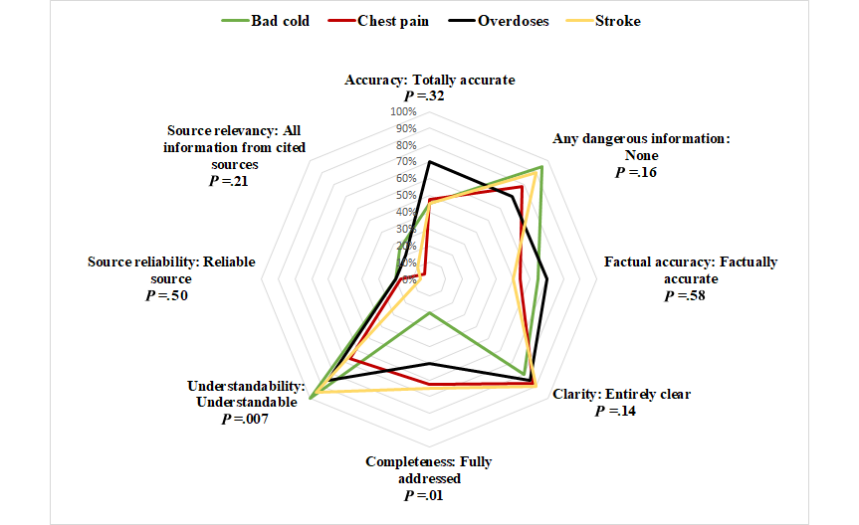
Comparison of condition-specific performance across different domains.

We depict the performance of the chatbots using radar charts, as in Figures 1-3, where the 8 domains are organized radially. Performance scores range from the best (100%) at the outer edge to the worst (0%) at the center. The further out the point of the polygon is, the better the chatbots perform in that domain. The outermost point in each domain represents the proportion of responses that the faculty reported as the best (totally correct, accurate, or complete) response from the scoring rubric.

### Comparative Analysis of Chatbot Performance

Each of the 4 chatbots was scored for 10 clinical questions across 8 domains by 5 EM faculty for 400 assessments for each chatbot. Figure 1 displays the overall performance of the 4 chatbots across the 10 medical conditions in 8 domains. Together, the 4 chatbots had the best performance in clarity and understandability (both 85%), intermediate performance in accuracy and completeness (both 50%), and poor performance (10%) for source relevance and reliability (most unreported).

Figure 2 compares the performance of the chatbots among the 8 studied domains. ChatGPT, Google Bard, and Claud AI had similar performance across 6 out of 8 domains. Only Bing AI performed better with more relevant and reliable sources. FKGL was 7.7-8.9 grade for all chatbots, except ChatGPT at 10.8, which were all too advanced for average emergency patients.

### Condition-Specific Performance (Serious vs Benign Conditions)

We compared the performance of the chatbots across a spectrum of seriousness of medical conditions. “Bad cold” was the least serious of the 10 conditions, and chest pain, overdose, and stroke were the most serious of the questions consumers may pose. Figure 3 shows significant variability in performance between serious and more benign conditions, where there was a significant difference in completeness (*P*=.01) and understandability (*P*=.007). This illustrates that chatbots are not consistent in their performance for different domains of “correctness,” but also not consistent for serious versus benign complaints. It is, therefore, important, not only to assess the chatbots’ performance across conditions and domains but also to consider the seriousness of the queried conditions. The consequences of misinformation (omissions or dangerous information) are potentially greater for more serious conditions.

### Safety and Dangerousness

**Figure 4:**
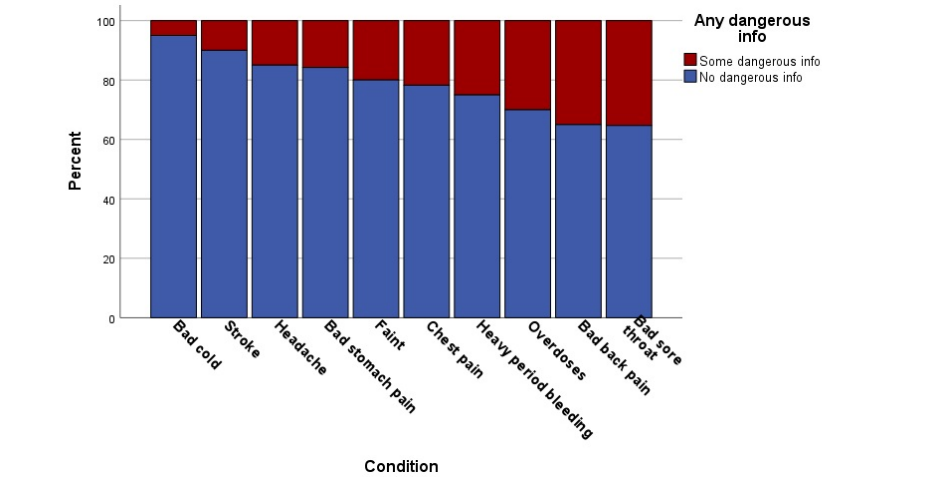
Comparison of safety and dangerousness across different medical conditions.

We compared the proportion of chatbot responses for each of the 10 conditions that were “dangerous,” in Figure 4. Examples included starting cardiopulmonary resuscitation (CPR) before any pulse check, moving a person who has fainted to “fresh air to help provide oxygen” without any assessment of potential injury or scene safety (carbon monoxide exposure), and use of a defibrillator without starting CPR while awaiting the device.

Overall, we found no statistically significant difference between chatbot responses on this dangerousness metric (Figure 2). Chatbots provided some dangerous information in all cases, from 5% for bad cold to 35% for both bad sore throat and bad back pain, with the most dangerous information (*P*=.03 for bad sore throat, and *P*=.04 for bad back pain, compared to the other 8 conditions). The other 8 conditions were statistically similar, yet all included minor to moderate proportions of dangerous information.

### Information Accuracy and Completeness

We measured the accuracy of chatbot responses in 2 ways. First, we asked EM faculty to report what proportion of the total information contained in the responses was accurate by percentage quartiles. Second, we asked them to identify and quantify (none, 1, 2, or more than 2) major and minor inaccuracies.

**Figure 5:**
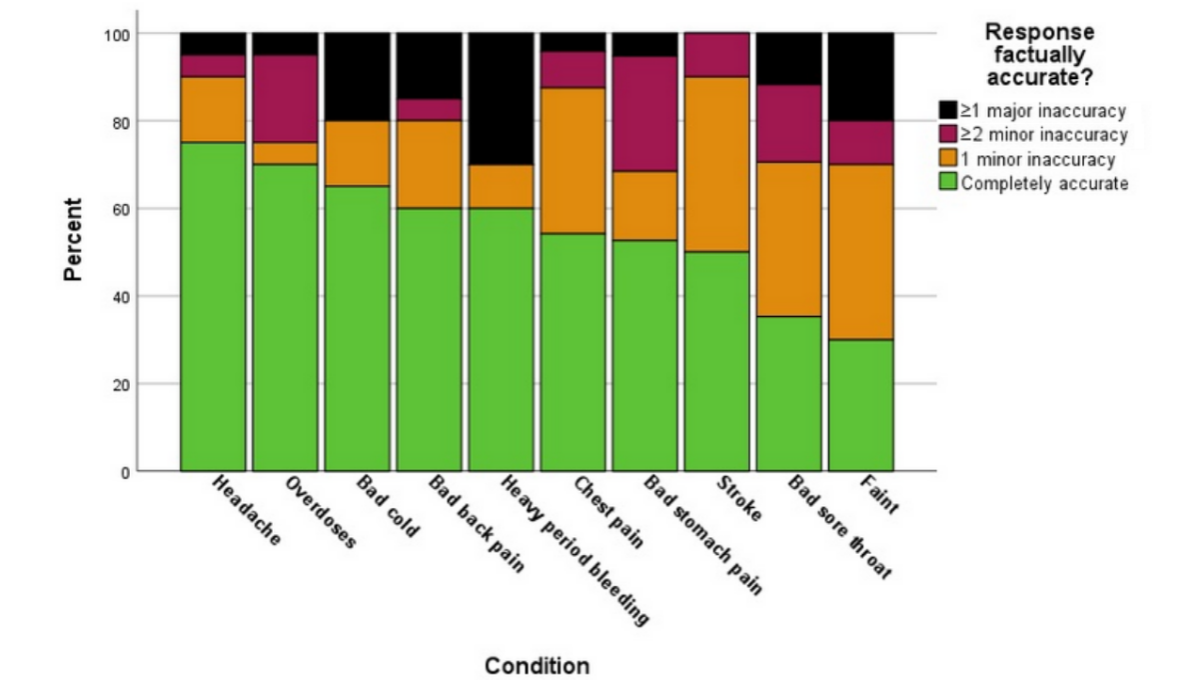
Comparison of factual accuracy across different medical conditions.

Figure 5 reports the accuracy of the 10 conditions from most (on the left of the figure) to least (on the right). Note again that there is significant variation by condition, as we showed above in Figure 3 (severity of condition) and Figure 4 (dangerousness).

For percentage accuracy across the 10 conditions, the “completely accurate” response proportion ranged from a high of 70% for headache and overdose, to a low of 30% for fainting. Conditions with a mere 25% accuracy included sore throat and fainting. Other conditions (back pain, stomach pain, period bleeding, stroke, cold, and chest pain) were intermediate in percentage accuracy. Comparing the 10 conditions, there were statistically significant differences between them in percentage accuracy (*P*=.01; using Monte Carlo simulations with 10,000 replications—99% CI 0.008-0.013), and major or minor factual accuracy (*P*=.01).

Because the order of conditions from most to least accurate, by the alternate percentage by quartile measure, closely matched the order of Figure 5, we elected not to present this percentile data.

As depicted in Figure 2, the aggregate measure of completeness across all 10 questions was similar and ranged from 50% to 60% for each of the 4 chatbots (*P*=.85). However, completeness of response did vary depending on the clinical condition (*P*=.04), from a high of 79% for stomach pain to a low of 20% for bad cold. The responses that were most deficient, as defined by missing more than 2 important pieces of information, varied from a low of 6% (bad sore throat) to a high of 40% (bad cold). The responses from all 10 conditions were judged by some faculty to be missing 1-2 pieces of important information in up to 20% of scores, while 5%-20% of scores identified more than 2 missing pieces of information. None of the 10 medical condition responses were judged by the faculty to contain all necessary information.

### Source Reliability and Relevance

**Figure 6:**
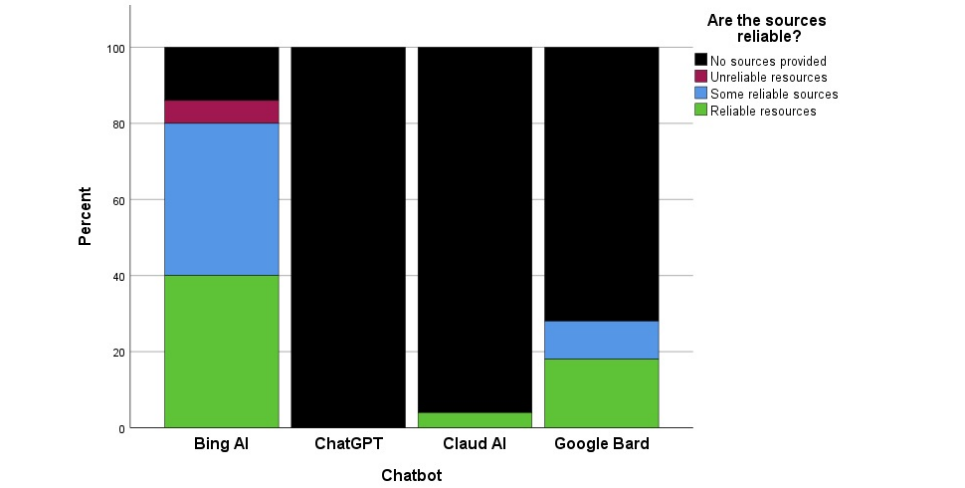
Comparison between 4 chatbots for the domain of source reliability.

As seen graphically in Figure 2, source reliability and source relevance were outliers from the other 6 domains. As shown in Figure 6, ChatGPT was a further outlier from the other chatbots (*P*<.001), providing essentially no sources for its responses, while Google Bard was slightly better than Claud AI (*P*=.003). Only Bing AI provided what the EM faculty judged as generally reliable sources for a majority of responses to the clinical queries. We found similar results for both domains of source reliability and source relevance, so only the former is presented here.

### Readability

**Figure 7:**
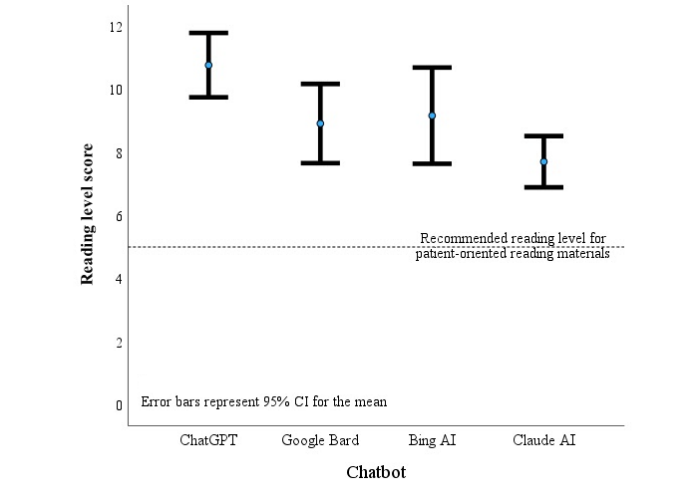
Comparison of mean and 95% CI (error bars) for reading level per Microsoft Word Flesch-Kincaid Grade Level (FKGL) score for 4 chatbots.

We processed all 10 clinical query responses from each chatbot through the embedded readability statistics tool in Microsoft Word (FKGL) to calculate a global grade reading level for each chatbot. Table 1 presents the distribution of grade level and chatbot average. The mean grade level for Google Bard, Bing AI, and Claude AI was 7.7-9.16, with ChatGPT scoring 10.76 grade level. As shown in Figure 7, ChatGPT scored higher than Claude AI (*P*=.003). The grade level across all 4 chatbots was statistically similar as the error bars overlapped (Figure 7). Within any of the 3 chatbots with lower overall grade-level reading scores, the level varied from a low of 5.9 to a high of 12.4. Of the 40 scores, the proportion of chatbot responses for all questions or conditions at or below each grade level was 0% (5th or below), 5% (6th), 15% (7th), 32.5% (8th), 52.5% (9th), 65% (10th), 80% (11th), and 90% at or below 12th grade.

Therefore, for the fifth-grade level recommended by the AMA for patient-oriented reading materials, all of the chatbot responses failed this standard. For the average reading level of the sixth grade for Medicaid-insured patients, the chatbot responses failed 95% of the time. Finally, the chatbots failed the US National Institutes of Health recommendation of eighth grade for reading materials 67.5% of the time [21,37].

**Table 1:** Emergency care responses to 10 clinical queries from four chatbots—FKGL^a^ scores from Microsoft Word.

Medical condition	Chatbots, FKGL score					Total, mean (SD)
	ChatGPT	Google Bard	Bing AI	Claude AI		
Chest pain	9.8	8.5	10	8.2	9.13 (0.91)	
Stroke	8.6	7.5	6.9	7	7.50 (0.78)	
Headache	10.9	9.8	9.8	7.1	9.40 (1.62)	
Sore throat	10.7	8.2	6.3	7.6	8.20 (1.85)	
Stomach pain	12.1	12.3	11.7	8	11.03 (2.03)	
Back pain	12.4	7.6	11.3	7.2	9.63 (2.61)	
Faints	8.5	6.3	6	5.9	6.68 (1.23)	
Period bleeding	11.4	10.3	9.8	10.3	10.45 (0.68)	
Bad cold	12.4	10.3	11.3	7.6	10.40 (2.05)	
Overdose	10.8	8.3	8.5	8.1	8.93 (1.26)	
Total, mean (SD)	10.76 (1.43)	8.91 (1.75)	9.16 (2.13)	7.70 (1.14)	N/A^b^	

^a^FKGL: Flesch-Kincaid Grade Level.

^b^N/A: not applicable.

## Discussion

### Principal Findings

We found that 4 chatbots had significant deficiencies in their performance compared to the opinions of 5 board-certified EM faculty and compared to optimal information drawn from respected mainstream EM texts and reliable websites. Problem areas included inadequate medical or scientific accuracy, incomplete information, dangerous information, and lack of source information.

The 4 chatbots were good and statistically similar in performance in clarity and understandability of presentation (to the educated EM faculty), intermediate in accuracy and completeness, but performed poorly for source disclosure and relevance. All 4 provided what the EM faculty considered dangerous advice with similar frequency. The grade level of English language presentation was above recommended norms for emergency patients (fifth to eighth-grade level) for all of them, with ChatGPT having the highest and, therefore, potentially least user-friendly, grade-level assessment (10th grade).

### Dangerous Information or Patient Safety

Because of the high stakes inherent in chatbot queries for emergency conditions, the authors were concerned about the potential for dangerous errors of omission and commission within the chatbot answers. These concerns were validated. There was wide variation in the accuracy of information depending on the clinical query and by chatbot. As all the chatbots had similar rates of dangerous advice and omissions of critical actions for some questions, we did not find any to be superior or inferior to others. We conclude, then, that chatbots may, if at all, be useful for minor conditions that are low risk (perhaps common cold) but should not be used for many EM complaints with potential for serious consequences and complications (chest pain, stroke, and overdose).

### Nonscientific Information

Another aspect of the veracity of chatbot responses is the degree to which they contain nonsensical or irrelevant information, without a scientific evidence base. Although our study design did not specifically ask the EM specialists to quantify these, we noted that responses contained such examples—gargling to relieve sore throat, using a heating pad for abdominal pain, eating vitamin C-rich foods to “boost your immune system” (multiple mentions of this), and “stay warm, as cold air can worsen sore throat, and avoid strong odors.”

Misinformation may be directly harmful. A recent report from the US Surgeon General and the US Department of Health and Human Services defines misinformation as—“Information that is false, inaccurate, or misleading according to the best available evidence at the time.” This continues—“...may cause people to make decisions that could have dangerous consequences for their health” [38]. Among our chatbot responses was the unscientific advice to use a heating pad for abdominal pain without any duration of application, which may delay evaluation for time-sensitive surgical conditions such as appendicitis.

The EM faculty also noticed many instances of vague advice that may not be understood by laypersons. Examples include “support the person’s breathing,” which may need “triage” to a stroke center, and references to “neurologic symptoms” or “other concerning symptoms” relating to headache.

### Reading Level and Understandability

For the specific use of chatbots in EM, readability or understandability is critical, perhaps beyond its importance for general medicine. Regardless of accuracy and completeness, if the consumer cannot readily comprehend the information from the chatbot query, this system of real-time health education fails on a fundamental level. Three of the chatbots had average responses written at the 7th to 9th grade level, with ChatGPT statistically higher at almost the 11th grade average. Even the “better” performing chatbots had some answers written at the 10th, 11th, and 12th grade levels. The lowest grade was grade level 5.9 and only 6 of the 40 answers were written at or below the seventh grade level (see Table 1). The authors conclude that these answers exceed the reading capability recommended in previous literature and US government recommendations and, therefore, may be unsuitable for potential emergency medical conditions [19-21,37].

### Spectrum of Queries From Not Likely Dangerous to Life-Threatening and Purposeful Spectrum

We chose 10 questions for the chatbots and EM faculty to reflect some of the most common presenting complaints to American EDs [22,39]. We purposely chose conditions with both benign and serious possibilities and hoped the responses would cover both ends of the acuity spectrum, and provide sound information for when, and in what time course, to seek further care. We found these features only inconsistently in each of the chatbots, with some queries containing such information, and some devoid of it. None of the chatbots performed consistently well on this measure.

### Standardized Study Reporting Framework and Assessment Tools are Lacking

We searched for, and then recognized, that there are no standard tools to evaluate health advice generated by chatbots. Hence, we derived our tool from recent papers that studied AI medical advice in other settings [7,8,17,30-34], and the expert opinions of EM faculty. We used both qualitative (minor and major missing information) and quantitative methods (percent of responses that were correct) to judge the chatbots.

With the increasing reliance of both physicians and patients on the internet for health advice, previous authors have stressed the importance of a standardized format for reporting AI-related health advice. Huo et al [29] wrote of their concern that these chatbots are a risk to patient safety. Further, they wrote, and we agree, that chatbot information needs accuracy, and must avoid false and misleading information or sources. These authors have convened an international group of stakeholders to address the importance of developing reporting standards for studies of chatbots used for health advice [29].

### Comparisons to Prior Work

Our paper’s methodology drafts from previous work, and substantially expands and improves on previous assessments of AI health care information. Recent publications have similarly reported that chatbots are not accurate enough to be used for emergency care. A study in pediatrics concluded that ChatGPT-4 appropriately advised to call emergency services in only 54% of cases and gave accurate first aid instructions in 45%. This paper concluded that its use as an “emergency support tool is questionable” [40]. A second paper showed 3 chatbots had similar but flawed performance to 176 questions from Reddit posed by actual adult patients. These authors divided the questions into “emergency” and “non-emergency,” and they reported the chatbots miscategorized 12%-35% of their cases. This study did not, like ours, evaluate the actual response and instructions, but only whether the question posed an emergency. Our study, then, goes beyond mere categorization, to assess the accuracy of the chatbot responses [41].

A recent paper from *JAMA Oncology* shared some of our methodologies; this paper extended those methods and tailored our evaluation to EM. The *JAMA Oncology* paper used a DISCERN tool (published in 1999) to evaluate information on 4 common cancers, and graded accuracy, quality, uncertainty, and reliability, on a 1-5 Likert scale [42]. We refined and expanded this by identifying 8 logical domains of information to assess the chatbot responses. We did not use a Likert scale purposely to limit subjectivity. Instead, we used specific percentages and numbers of inaccurate pieces of information, as judged by content experts in EM.

The previous paper’s scoring was not apparently done by content experts. We had our EM experts compare the chatbot responses to “correct” answers drawn from reputable and widely used sources. The *JAMA Oncology* authors similarly assessed the readability of the chatbot responses and found overall 11-12 grade reading level, was certainly too high for the average emergency patient [42].

A more recent grading tool for web-based medical education information was published in 2016 by ALIEM and shared some of our domains for evaluation—accuracy, use, evidence base, and reference-quality [30]. They used a 1-7 Likert scale and similarly had multiple (8) experts evaluate each of the online resources. As these were geared toward physician learners, they did not evaluate readability.

### Categories and Examples of Response Deficiencies

The chatbot responses had both categorical and individual deficiencies. The major categories were important omissions of critical information, advice to act with insufficient situational information, absence of information regarding when and how to access health care, vague or technical language not expected to be understandable by laypeople, and extraneous information without scientific support.

There were components of some responses that were considered “omissions,” but were so important that the omissions were also considered “dangerous actions.” Examples include omitting a pulse check with chest pain, and yet advising to start CPR, and omitting any mention of lack of need or efficacy of antibiotics for the common cold. Other overtly dangerous actions were advising the use of a defibrillator without directing anyone to start CPR, and advice to move a patient who fainted to fresh air without checking for any injury. Some responses also made no mention of pregnancy, either ectopic or intrauterine, among the advice for vaginal bleeding. Finally, the advice for sore throat omitted any mention of symptoms of airway obstruction, like stridor.

As an example of dangerous advice, Bing (now called “Copilot”) responded to the question of chest pain with reasonable advice to call 911, don’t drive yourself, chew aspirin, and take nitroglycerin if already prescribed. The introduction said “it’s essential to not self-diagnose...” but then said “...Begin CPR (Cardiopulmonary Resuscitation): If the person is having a heart attack start hands-only CPR (emphasis added)...Push hard and fast on the person’s chest for 100 to 120 compressions per minute. If an automated external defibrillator (AED) is available, follow the device instructions.”

While this last advice is also correct, the answer requires the prompter to, in fact, self-diagnose a heart attack. It then does not state that the patient needs to become unconscious and have no pulse, yet advises to start CPR.

Advice to consumers about when and how to seek medical care was inconsistent and vague. We recognize that EM faculty would have a bias toward expecting the inclusion of “red-flag” symptoms. For example, for severe or persistent headaches, advice to go to the emergency room for “concerning neurological symptoms” is too vague to be useful. Conversely, some responses mentioned serious conditions to watch for but omitted any advice for common conditions with the same complaint. For example, Bing AI provided specific signs to watch for stroke or meningitis but did not give advice about migraine or tension headaches. Finally, 1 chatbot advised in the possible stroke information, for the reader to start CPR, without assessing if the patient was unconscious or pulseless.

At the time of the study, 1 chatbot, Bing AI, provided better source information than the other 3. Chat-GPT, Claude AI, and Google Bard, when asked, “Please list all sources of information you referenced,” they generally responded, “I don’t have direct access to my training data or know where it came from. I was trained on a mixture of licensed data, data created by human trainers, and publicly available data.”

Bing AI, on the other hand, provided references to “learn more,” which some faculty interpreted as providing the sources of information for the answer. In addition, some of Bing AI’s components of the answers were themselves, hyperlinks from which the information was derived and enabled the user to directly access some sources. Some “learn more” links were to reliable sources like the National Health Service in the United Kingdom, the USA Centers for Disease Control and Intervention, and Mayo and Cleveland Clinics, while others were clearly not authoritative. For example, Bing AI listed medical news sources like MSNBC, proprietary information from various urgent cares, computer resources like Microsoft Start, and layperson sites like Verywell Health and WikiHow. This combination of some reliable sources and embedded hyperlinks from Bing AI explains why it was judged by the experts as superior to the other 3 in this 1 domain.

### Strengths and Limitations

This paper is the first, to our knowledge, to compare and contrast multiple chatbots for accuracy, readability, and potential misinformation of answers to patient questions, as judged by multiple academic faculty content experts in the field of EM. As the decision to seek emergency care is high-stakes and high-cost, the authors felt that this level of rigor was appropriate.

This study has several limitations. First, while we conducted a pilot test of the scoring sheet or methodology on 3 of the included clinical conditions (bad cold, fainting, and chest pain) and refined the scoring sheet and methodology with the content experts, we did not pilot all 10 queries. Second, despite including definitions for each question in the survey, there is still a risk that the EM faculty might interpret the questions differently. Third, we did not examine the causes of discrepancies among the faculty in their assessments of source relevance and reliability. Some raters interpreted, “learn more,” at the end of the Bing AI chatbot response to be a reporting of sources from which the answers were derived, and some did not. We did not further study this variability in interpretation. Fourth, we did not do a cluster analysis for the 5 raters, leaving the potential that some were inherently more severe graders of AI than others. Fifth, although we derived our clinical questions from among the most common ED chief complaints [22], beyond purposely choosing a mixture of benign and potentially serious conditions, we did not test actual patient responses or actions to the chatbot information. Sixth, we studied the free versions of the chatbots available at the time. Certainly, this is a moving landscape, and there have been updates and improvements in the products since this study was conducted. However, one can only study what is available, and future studies should examine more current and potentially capable versions. Seventh, we chose only 10 questions as a compromise between comprehensiveness and feasibility. Each faculty expert performed 320 assessments (10 questions × 4 chatbots × 8 domains). This number of questions may be insufficient to comprehensively judge the performance of each chatbot. Finally, we did not test the “right answers” derived from reputable medical resources for the 5 faculty members to judge if they considered them truly, “correct.” This would have been an appropriate control. Future studies should include this methodology to separate out potential deficiencies in chatbot responses from general variability in expert opinion.

### Future Directions

We believe that GenAI will have an impact on social determinants of health, either by making access to accurate health information more accessible to the masses, or rapidly disseminating potentially inaccurate health information to patients with limited access to in-person medical care. For this reason, and because our findings and others have called into question the accuracy of GenAI health information, we believe further research and policy decisions should focus on appropriate regulation by governments and industry.

We would no sooner accept approval of a new pacemaker or orthopedic implant without FDA clearance than we would allow wide access to incomplete, dangerous, nonscientific information to guide patients’ health care decisions. The authors recognize the enormous challenge of regulating words produced by these systems. Nevertheless, we would be remiss in not calling for progress in this area.

Both the European Parliament and the US federal government, as well as China’s Ministry of Industry and Information Technology have recognized the need for regulation of AI and put forth draft regulations. As these efforts are just beginning and very fluid, we direct the reader to these references for further information [43-46].

### Conclusions

AI chatbots have important deficiencies for EM patient advice, despite their consistent performance. Advice for when to seek urgent or emergent care is frequently incomplete and inaccurate, and patients may be unaware of misinformation. Sources of information are not generally disclosed. Patients who use AI to guide their health care assume potential risks. The use of AI chatbots for health information should be subject to further research, refinement, and regulation. We strongly recommend proper medical consultation to prevent potential adverse outcomes. Finally, we call for further validation of scoring tools and the development of literature reporting guidelines.

## Appendix

Multimedia Appendix 1 Research prompts.

Multimedia Appendix 2 Chatbot log.

Multimedia Appendix 3 Chatbot 10 right answers.

Multimedia Appendix 4 Google scoring form.

## Abbreviations

AC1: Agreement Coefficient 1
AI: artificial intelligence
AMA: American Medical Association
CPR: cardiopulmonary resuscitation
ED: emergency department
EM: emergency medicine
FDA: US Food and Drug Administration
FKGL: Flesch-Kincaid Grade Level
GenAI: generative artificial intelligence
LLM: large language model
NCHS: National Center for Health Statistics

## Data Availability

The datasets generated and analyzed during this study are available from the corresponding author on reasonable request.

## References

[1] Hoffman M (2020). What is a chatbot + how does it work? The ultimate guide. Zendesk.

[2] Joseph A, Eapen NG, Pillai AS, Tedesco R (2023). Conversational agents and chatbots: current trends. Machine Learning and Deep Learning in Natural Language Processing.

[3] Laymouna M, Ma Y, Lessard D, Schuster T, Engler K, Lebouché Bertrand (2024). Roles, Users, Benefits, and Limitations of Chatbots in Health Care: Rapid Review. J Med Internet Res.

[4] (2020). What is a chatbot?. IBM.

[5] Metz C, Grant N (2023). Google updates bard chatbot with ‘Gemini’ A.I. as it chases ChatGPT. The New York Times.

[6] Mehdi Y (2023). Reinventing search with a new AI-powered microsoft bing and edge, your copilot for the web. Official Microsoft Blog.

[7] Kung TH, Cheatham M, Medenilla A, Sillos C, de Leon L, Elepaño C, Madriaga M, Aggabao R, Diaz-Candido G, Maningo J, Tseng V (2023). Performance of ChatGPT on USMLE: potential for AI-assisted medical education using large language models. PLOS Digit Health.

[8] Yeo YH, Samaan JS, Ng WH, Ting P, Trivedi H, Vipani A, Ayoub W, Yang JD, Liran O, Spiegel B, Kuo A (2023). Assessing the performance of ChatGPT in answering questions regarding cirrhosis and hepatocellular carcinoma. Clin Mol Hepatol.

[9] Liu M, Okuhara T, Chang X, Shirabe R, Nishiie Y, Okada H, Kiuchi T (2024). Performance of ChatGPT across different versions in medical licensing examinations worldwide: systematic review and meta-analysis. J Med Internet Res.

[10] Reddy S (2023). Evaluating large language models for use in healthcare: a framework for translational value assessment. Inform Med Unlocked.

[11] Wang X, Cohen RA (2022). Health Information Technology Use Among Adults: United States.

[12] Can GenAI help make health care affordable? Consumers think so. Deloitte US.

[13] Shahsavar Y, Choudhury A (2023). User intentions to use ChatGPT for self-diagnosis and health-related purposes: cross-sectional survey study. JMIR Hum Factors.

[14] Hanebutt R, Mohyuddin H (2023). The digital domain: a "Super" social determinant of health. Prim Care.

[15] (2023). Digital access: a super determinant of health. Substance Abuse and Mental Health Services Administration.

[16] Johnson D, Goodman R, Patrinely J, Stone C, Zimmerman E, Donald R, Chang S, Berkowitz S, Finn A, Jahangir E, Scoville E, Reese T, Friedman D, Bastarache J, van der Heijden Y, Wright J, Carter N, Alexander M, Choe J, Chastain C, Zic J, Horst S, Turker I, Agarwal R, Osmundson E, Idrees K, Kieman C, Padmanabhan C, Bailey C, Schlegel C, Chambless L, Gibson M, Osterman T, Wheless L (2023). Assessing the accuracy and reliability of AI-generated medical responses: an evaluation of the Chat-GPT model. Res Sq.

[17] Samaan JS, Yeo YH, Rajeev N, Hawley L, Abel S, Ng WH, Srinivasan N, Park J, Burch M, Watson R, Liran O, Samakar K (2023). Assessing the accuracy of responses by the language model ChatGPT to questions regarding bariatric surgery. Obes Surg.

[18] Jin Q, Leaman R, Lu Z (2023). Retrieve, summarize, and verify: how will ChatGPT affect information seeking from the medical literature?. J Am Soc Nephrol.

[19] Powers RD (1988). Emergency department patient literacy and the readability of patient-directed materials. Ann Emerg Med.

[20] Rooney MK, Santiago G, Perni S, Horowitz DP, McCall AR, Einstein AJ, Jagsi R, Golden DW (2021). Readability of patient education materials from high-impact medical journals: a 20-year analysis. J Patient Exp.

[21] Weiss BD, Blanchard JS, McGee DL, Hart G, Warren B, Burgoon M, Smith KJ (1994). Illiteracy among Medicaid recipients and its relationship to health care costs. J Health Care Poor Underserved.

[22] Arvig MD, Mogensen CB, Skjøt-Arkil H, Johansen IS, Rosenvinge FS, Lassen AT (2022). Chief complaints, underlying diagnoses, and mortality in adult, non-trauma emergency department visits: a population-based, multicenter cohort study. West J Emerg Med.

[23] Altamimi I, Altamimi A, Alhumimidi AS, Altamimi A, Temsah M (2023). Snakebite advice and counseling from artificial intelligence: an acute venomous snakebite consultation with ChatGPT. Cureus.

[24] Fahy E, Hardikar R, Fox A, Mackay S (2014). Quality of patient health information on the internet: reviewing a complex and evolving landscape. Australas Med J.

[25] Liu J, Zhang Y, Kim Y (2023). Consumer health information quality, credibility, and trust: an analysis of definitions, measures, and conceptual dimensions.

[26] Eysenbach G, Powell J, Kuss O, Sa ER (2002). Empirical studies assessing the quality of health information for consumers on the world wide web: a systematic review. JAMA.

[27] Robillard JM, Jun JH, Lai JA, Feng TL (2018). The QUEST for quality online health information: validation of a short quantitative tool. BMC Med Inform Decis Mak.

[28] Breckons M, Jones R, Morris J, Richardson J (2008). What do evaluation instruments tell us about the quality of complementary medicine information on the internet?. J Med Internet Res.

[29] Huo B, Cacciamani GE, Collins GS, McKechnie T, Lee Y, Guyatt G (2023). Reporting standards for the use of large language model-linked chatbots for health advice. Nat Med.

[30] Chan TM, Grock A, Paddock M, Kulasegaram K, Yarris LM, Lin M (2016). Examining reliability and validity of an online score (ALiEM AIR) for rating free open access medical education resources. Ann Emerg Med.

[31] Coskun B, Ocakoglu G, Yetemen M, Kaygisiz O (2023). Can ChatGPT, an artificial intelligence language model, provide accurate and high-quality patient information on prostate cancer?. Urology.

[32] Rahsepar AA, Tavakoli N, Kim GHJ, Hassani C, Abtin F, Bedayat A (2023). How AI responds to common lung cancer questions: ChatGPT vs Google Bard. Radiology.

[33] Lahat A, Shachar E, Avidan B, Glicksberg B, Klang E (2023). Evaluating the utility of a large language model in answering common patients' gastrointestinal health-related questions: are we there yet?. Diagnostics (Basel).

[34] Morath B, Chiriac U, Jaszkowski E, Deiß C, Nürnberg H, Hörth K, Hoppe-Tichy T, Green K (2023). Performance and risks of ChatGPT used in drug information: an exploratory real-world analysis. Eur J Hosp Pharm.

[35] Klein D (2018). Implementing a general framework for assessing interrater agreement in Stata. Stata J.

[36] Do you need IRB review?. UCI Office of Research.

[37] Weiss BD (2003). Health literacy: a manual for clinicians. American Medical Association Foundation and American Medical Association.

[38] (2024). Health misinformation. U.S. Department of Health and Human Services.

[39] (2023). Estimates of emergency department visits in the United States, 2016-2021. National Center for Health Statistics.

[40] Bushuven S, Bentele M, Bentele S, Gerber B, Bansbach J, Ganter J, Trifunovic-Koenig M, Ranisch R (2023). "ChatGPT, Can You Help Me Save My Child's Life?" - diagnostic accuracy and supportive capabilities to lay rescuers by ChatGPT in prehospital basic life support and paediatric advanced life support cases - an in-silico analysis. J Med Syst.

[41] Zúñiga Salazar G, Zúñiga D, Vindel CL, Yoong AM, Hincapie S, Zúñiga AB, Zúñiga P, Salazar E, Zúñiga B (2023). Efficacy of AI chats to determine an emergency: a comparison between OpenAI's ChatGPT, Google Bard, and Microsoft Bing AI Chat. Cureus.

[42] Pan A, Musheyev D, Bockelman D, Loeb S, Kabarriti AE (2023). Assessment of artificial intelligence Chatbot responses to top searched queries about cancer. JAMA Oncol.

[43] (2023). Executive order on the safe, secure, and trustworthy development and use of artificial intelligence. The White House.

[44] Artificial intelligence 2023 legislation. National Conference of State Legislatures.

[45] Ye J (2024). China issues draft guidelines for standardising AI industry. Reuters.

[46] (2023). EU AI act: first regulation on artificial intelligence. Topics European Parliament.

