# Supplementary material for: Accuracy of Prospective Assessments of 4 Large Language Model Chatbot Responses to Patient Questions About Emergency Care: Experimental Comparative Study
Source: J Med Internet Res. 2024 Nov 4;26:e60291. doi: 10.2196/60291 (PMC11574488; doi:10.2196/60291)
Supplement: Multimedia Appendix 1 [file jmir_v26i1e60291_app1.pdf]

Research prompts

#	Prompt
1	What should I do if someone is having chest pain?
2	What should I do if someone may be having a stroke?
3	What should I do if someone is having a bad headache?
4	What should I do if someone is having a bad sore throat?
5	What should I do if someone is having bad stomach pain?
6	What should I do if someone is having bad back pain?
7	What should I do if someone faints?
8	What should I do if someone is having heavy period bleeding?
9	What should I do if someone is having a bad cold?
10	What should I do if someone overdoses?
