# Supplementary material for: Accuracy of Prospective Assessments of 4 Large Language Model Chatbot Responses to Patient Questions About Emergency Care: Experimental Comparative Study
Source: J Med Internet Res. 2024 Nov 4;26:e60291. doi: 10.2196/60291 (PMC11574488; doi:10.2196/60291)
Supplement: Multimedia Appendix 3 [file jmir_v26i1e60291_app3.pdf]

Chatbot 10 Right Answers

1) What should I do if someone is having chest pain?

Chest pain has a wide range of characteristics as well as causes. The causes of chest pain may vary depending on characteristics of the chest pain, a person's medical history, and a person's risk factors. Many people associate chest pain with heart attacks; however, there are many other causes. Causes of chest pain may include non-cardiac causes including digestive, lung related, muscle and bone, or anxiety causes. There are various tests in the emergency room that could help narrow the possible causes of chest pain and thus if there is concern, immediate medical attention should be sought out for assessment.

If someone describes their chest pain as a sudden onset of crushing or pressure like pain, worsening with activity, shortness of breath, associated arm pain, and sweating, these are very concerning symptoms of a heart attack, and you should call 911 and go to the ER immediately. A heart attack may also present with more subtle symptoms including chest pain of other types, with some other features of nausea, vomiting, dizziness, weakness or fast or irregular heartbeat (palpitations). The emergency department will be able to evaluate heart attack, as well as other causes, with tests including ECG, blood tests evaluating heart damage, chest Xrays, and CT scans. If you are concerned about your symptoms seek medical attention right away.[1–3]

2) What should I do if someone may be having a stroke?

If you believe someone may be having a stroke you should seek immediate attention and call 911. Signs to look out for include facial droop/numbness on one side, slurred speech or difficulty speaking, one-sided weakness of an arm or leg or both, sudden severe headache, or loss of balance (unable to stand, or falling to one side). Factors that put someone at higher risk of a stroke include smoking, high blood pressure, diabetes, atrial fibrillation, high cholesterol, and family history of stroke. Other risk factors include older age, excessive alcohol history, high fat diet and low level of activity, and obesity. Time is one of the most important factors in outcome, so call 911 to be taken to a hospital right away. Do not drive yourself. At the hospital and Emergency Department, physicians will be able to diagnose the type of stroke and treat it.

It may be helpful to keep the acronym “FAST” in mind when looking for symptoms of stroke. “F” standing for Face, asking the person to smile, and looking for any one-sided facial droop. “A” standing for Arms, asking the person to close their eyes, raise both arms with palms up for 10 seconds, and looking for an arm drifting downward. “S”

stands for Speech, asking the person to repeat a phrase and evaluating for any slur in speech or difficulty reproducing the phrase. “T” stands for Time as the most important aspect in a suspected stroke is getting medical attention as quickly as possible. It is important to be seen by the doctor right away, as treatment is necessary as early as possible and within 4.5 hours, depending on the type of stroke.[4–6]

3) What should I do if someone is having a bad headache?

There are many different types and causes of headaches. If someone is having a bad headache, ask them what other symptoms they are having. If someone has a sudden severe headache with difficulty seeing, speaking, weakness in one part of the body, numbness or trouble with balance/falling over, or decreased responsiveness, you should call 911 right away, as these may be symptoms of a bleeding stroke. If symptoms above are absent, but there is a severe headache that lasts more than 3 hours, with symptoms of a fever, stiff neck, or any confusion, you should also seek immediate attention in the emergency department, as these are symptoms of meningitis, an infection of the outside of the brain that needs immediate treatment with strong antibiotics to fight infection.[7]

There are many types of headaches that are less severe and emergent than those above. Primary headaches start from the brain or head and are not associated with underlying disease. They include cluster headaches, tension headaches, and migraines. Secondary headaches are associated with underlying disease such as sinusitis, an infection in the airspaces of the face, or the flu (a viral infection caused by the influenza virus, or other viruses).[8] If you suspect a bad headache is similar to these, there are different medications that may treat the symptoms. Over the counter medicines like ibuprofen and Tylenol help reduce the pain of the headache. Cold medicines can reduce congestion and help sinus headaches. If these don’t help and the headache persists, you should see a doctor for assessment and possible need for prescription medicines.[9]

If someone has a bad headache and has hit their head in the past few days, AND is taking blood-thinning medicines for their heart or for blood clots, or taking aspirin, they should see a doctor immediately.

4) What should I do if someone is having a bad sore throat?

Sore throats have many different causes including viruses (most common), bacteria (rarely), allergies, and smoking. Most commonly, a sore throat is caused by viruses such as cold or flu, and these usually go away on their own in a few days. They can be treated with over-the-counter cold medicines to reduce congestion and treat pain, like ibuprofen (Motrin) or acetaminophen (Tylenol).[10,11] Most sore throats are from viruses and not bacteria, and antibiotics don’t treat viral sore throats. So most sore

throats do not need antibiotics. Bacterial infections such as strep are much less common and may require antibiotics for resolution and to prevent other causes of sore throat include environmental causes such as allergies or smoking. Any kind of tobacco or marijuana smoking can cause sore throat.[12]
Since most sore throats are caused by viral infections, the most important thing to do for a bad sore throat is to treat the symptoms. Over the counter medicines including cough and cold medicine are helpful for reducing viral symptoms, as well as Tylenol for fevers and pain. Ice, popsicles, honey, and gargling of salt water may all be helpful in soothing a sore throat.[13] If a sore throat lasts for longer than a week or includes difficulty breathing, swallowing, fever more than 101.4 (38.0), blood in saliva, rash, swollen lymph nodes (glands) in the neck or muffling of voice, you should seek immediate attention.[12]

5) What should I do if someone is having bad stomach pain?

Stomach pains have many different causes. See a doctor immediately if you have bad stomach pain with bloody vomit, black (not brown) stools, unintended weight loss more than 5 pounds, distended or if the abdomen is stiff or rigid, and not soft when you push on it. Dark/black stools or dark vomit that looks like coffee grounds are signs of bleeding in your stomach from an ulcer or from another area in your intestines and require immediate attention. Risk factors of bleeding from a stomach ulcer include overuse of ibuprofen (Motrin), smoking, or infection with a stomach bacteria called H. Pylori. Other reasons for immediate medical attention is if the stomach pain moves up to the chest or arms, or has shortness of breath or sweating with it. These may be signs of heart attack.[14]
Most times stomach pain is not serious and is caused by excess stomach acid, gas, or constipation. Bad stomach pain may be relieved with over-the-counter medications that reduce stomach acid or gas. Simethicone helps with gas pains. Medicines that help reduce acid include H2 receptors, including famotidine (Pepcid AC) or ranitidine (Zantac). Others reduce the stomach acid pump, like omeprazole (Prilosec OTC).[15]
If you have had stomach pain that continues for more than a few days, see a primary care physician in the office who may do tests for H. Pylori, a stomach bacteria. If this test is positive, your primary care physician may prescribe antibiotics that would help this.

6) What should I do if someone is having bad back pain?

Bad back pain has many different causes including arthritis, muscle or ligament strain, bulging or ruptured disks, osteoporosis or inflammatory diseases. Back pain is commonly persistent and severe, even when it doesn’t come from a serious cause or injury. It is important to tell your primary care physician about your bad back pain to try to determine its cause and develop a treatment plan. They may offer anti-inflammatory medications, muscle relaxants, topical (applied to the skin) pain relievers, and rarely, for severe chronic pain, opioids, or antidepressants. Your primary care physician may

also refer you to physical therapy for strengthening/stretching of supporting back muscles that may help relieve pain.[16]

You should seek immediate attention if you have any signs of leg or arm weakness or numbness with severe back pain, especially if following a fall or injury. If there is back pain with difficulty walking due to leg weakness, loss of control of urine or stool (it leaks out without you realizing it) OR if you can't urinate for more than 12 hours even though you try, or if you haven't had any bowel movement in more than 3 days,, numbness of the area of your genitals and between them and your anus, these are signs of compression of spinal cord within the bones of your back, and requires immediate medical attention. If a person has severe back pain, along with fever and fatigue, this may be a spinal cord infection and requires immediate care for antibiotics.[16] In these situations, xrays CT or MRI, blood tests, and physical exam are necessary to determine the cause of back pain and the course of treatment.[17,18]

7) What should I do if someone faints?

If someone faints and does not awaken and feels normal within 30 seconds, you should call 911 or seek immediate medical attention. If the fainting person doesn't wake up within 30 seconds, feel for a pulse and check to see if they are breathing. If you do not feel a pulse, you should immediately initiate CPR (chest compressions) and call 911. If they are not breathing, you should immediately start rescue breathing with a bag-valve-mask apparatus if in the hospital, or mouth to mask ventilation and call 911. Fainting has many different causes including bad heart rhythms, low blood sugar, low blood pressure, blood loss, severe infections, heat or dehydration, or some medications. It is important to note the circumstances of a patient fainting to provide clues to the cause. These include new medicines, history of heart disease, recent severe illness, extreme heat, or symptoms prior to fainting.[19]

The potential cause of fainting will influence the potential treatments. If you suspect they may have fainted from heat exhaustion, cool the person down as much as you can (move indoors out of the sun or into air conditioned space) and provide them with water when they are awake and can drink. If the fainting may be due to low blood pressure, have the person lay flat on the ground (not sit or stand). This will help return blood flow to brain and keep them awake. People who faint, even if they wake up right away, should still be seen by a medical professional to try to find out why.[20,21]

8) What should I do if someone is having heavy period bleeding?

First ensure that she is not having signs of dangerous bleeding including pale or sweaty skin, dizziness, lightheadedness on standing, or fainting. If she has these symptoms, call 911. Heavy period bleeding may be due to hormone issues, birth control use, uterine fibroids or even cancer.[22] Heavy bleeding may not be from a

normal period, but instead a sign of pregnancy. If she is pregnant (or may be), and has heavy bleeding, go to the ER right away to be seen by a doctor.

The cause of heavy period bleeding depends on age, number of previous pregnancies and medical history. Make an appointment with a primary care physician or gynecologist (specialist in women's health and pregnancy) to discuss heavy period bleeding. During these appointments they may do blood tests, an internal vaginal exam, or ultrasound to find out the cause. Your primary care physician or gynecologist may recommend increasing iron in your diet that helps build blood, increase physical activity, or weight loss. Do not take ibuprofen (Motrin) or aspirin (and other NSAIDs) as they can increase the bleeding. Tylenol is ok, may help cramping pains, and does not increase bleeding. The doctor may start oral contraceptive (birth control pills) not to prevent pregnancy, but to restore your normal monthly hormone cycle that determines your periods.[23]

9) What should I do if someone is having a bad cold?

The symptoms of a cold last 7-10 days. Common symptoms include runny nose, cough, nose congestion, sore throat, or headaches (medlineplus.gov). There are many medicines you can buy at the drug store that may help. Tylenol (acetaminophen), Motrin (ibuprofen) both can help pain from sore throat or muscle aches (okay for children). You can take both together because they work in different ways. Use a decongestant nasal sprays (like Afrin) or a pill. The medicine pseudoephedrine (Sudafed) works very well to dry up your nose. In addition, take an antihistamine like loratadine (Claritin) or fexofenadine (Allegra) to control congestion and itching. Cough syrups only help a little, if at all, and prescription cough medicines are not really any better. So, you should take Tylenol or Motrin (or both), Claritin or Allegra (not both), and Sudafed to best manage your cold symptoms.

If these medications do not help, or the cold lasts more than 7 days, you should see a doctor. The doctor may prescribe an asthma inhaler if you are wheezing, and depending on your symptoms and vital signs, may order a chest Xray.[24]

Complications of a bad cold include pneumonia (lung infection), middle ear infections (especially in children), sinus infection, or asthma attacks. You are at higher risk for these if you are older than 50, have asthma, if you smoke tobacco or marijuana or vape. If you are immunocompromised by medical conditions or take medicines that suppress your immune system, you should consult a doctor and not wait 7 days.[25]

Wash your hands frequently, stay 6 feet away from other people if possible, and wear a mask if you're sick and have to be around other people. Stay home if you're sick. This may help keep others from getting sick. Get vaccinated against flu each year in the fall, against COVID, RSV and pneumonia consistently to reduce the risk of infection and severe disease.

Antibiotics do not fight the viruses that cause the common cold and are not necessary to treat it. Antibiotics only treat bacteria, which are never the cause of a common cold. You do not need to see a doctor to get antibiotics for your cold.

10) What should I do if someone overdoses?

Immediately call 911. Check to see if that person has a pulse in their neck (if you know how) and if they are breathing (look at the chest to see if it moves). If they do not have a pulse, start CPR by doing chest compressions at a rate of 100-120 beats/minute. Continue until paramedics arrive. If a patient is not breathing, give rescue breaths with a bag-valve-mask device in a hospital, or a mouth to mask device. If you suspect an overdose on opioids/opiates (heroin, fentanyl, "oxy") and have Narcan (naloxone), immediately give this in the nose/nostril to reverse the effects of opioids.[26] Anyone with a suspected overdose who is breathing too slow or too shallow (chest hardly moving) should get Narcan/naloxone in the nose right away. There is no danger to give Narcan/naloxone, even if the overdose is from something else.

Overdoses may come from many different drugs. Most overdoses from opioids or fentanyl make the patient stop breathing, leading to death if not treated with Narcan/naloxone within 5-10 minutes. However, overdoses may come from many different drugs. Seek immediate medical attention (call 911) for any overdose. Give information to the ER about what you suspect the overdose is from. Take any medication pill bottles, drug paraphernalia, or empty home cleaning products.[26] The ER only wants to help the overdose victim and won't contact the police.[27]

References

---

1. Outpatient evaluation of the adult with chest pain - UpToDate. Available from: https://www.uptodate.com/contents/outpatient-evaluation-of-the-adult-with-chest-pain?search=chest%20pain&source=search_result&selectedTitle=1~150&usage_type=default&display_rank=1#H3376492356 [accessed Aug 25, 2024]
2. Chest pain - Symptoms and causes. Mayo Clin. Available from: https://www.mayoclinic.org/diseases-conditions/chest-pain/symptoms-causes/syc-20370838 [accessed Aug 25, 2024]
3. Chest Pain. National Library of Medicine; Available from: https://medlineplus.gov/chestpain.html [accessed Aug 25, 2024]
4. Initial assessment and management of acute stroke - UpToDate. Available from: https://www.uptodate.com/contents/initial-assessment-and-management-of-acute-stroke?search=stroke&source=search_result&selectedTitle=1~150&usage_type=default&display_rank=1 [accessed Aug 25, 2024]
5. Stroke - Symptoms and causes - Mayo Clinic. Available from: https://www.mayoclinic.org/diseases-conditions/stroke/symptoms-causes/syc-20350113 [accessed Aug 25, 2024]

6. Stroke. National Library of Medicine; Available from: https://medlineplus.gov/stroke.html [accessed Aug 25, 2024]
7. Evaluation of headache in adults - UpToDate. Available from: https://www.uptodate.com/contents/evaluation-of-headache-in-adults?search=headache&source=search_result&selectedTitle=1~150&usage_type=default&display_rank=1#H1639554284 [accessed Aug 25, 2024]
8. Sinus headaches are usually migraines-Sinus headaches - Symptoms & causes. Mayo Clin. Available from: https://www.mayoclinic.org/diseases-conditions/sinus-headaches/symptoms-causes/syc-20377580 [accessed Aug 25, 2024]
9. Headache. National Library of Medicine; Available from: https://medlineplus.gov/headache.html [accessed Aug 25, 2024]
10. Sore throat - Symptoms & causes. Mayo Clin. Available from: https://www.mayoclinic.org/diseases-conditions/sore-throat/symptoms-causes/syc-20351635 [accessed Aug 26, 2024]
11. Sore throat - Diagnosis & treatment. Mayo Clin. Available from: https://www.mayoclinic.org/diseases-conditions/sore-throat/diagnosis-treatment/drc-20351640 [accessed Aug 26, 2024]
12. Evaluation of acute pharyngitis in adults - UpToDate. Available from: https://www.uptodate.com/contents/evaluation-of-acute-pharyngitis-in-adults?search=sore%20throat&source=search_result&selectedTitle=2~150&usage_type=default&display_rank=1#H31 [accessed Aug 26, 2024]
13. Sore Throat. National Library of Medicine; Available from: https://medlineplus.gov/sorethroat.html [accessed Aug 26, 2024]
14. Abdominal Pain. National Library of Medicine; Available from: https://medlineplus.gov/abdominalpain.html [accessed Aug 26, 2024]
15. Functional dyspepsia - Symptoms and causes. Mayo Clin. Available from: https://www.mayoclinic.org/diseases-conditions/functional-dyspepsia/symptoms-causes/syc-20375709 [accessed Aug 26, 2024]
16. Back pain - Symptoms and causes. Mayo Clin. Available from: https://www.mayoclinic.org/diseases-conditions/back-pain/symptoms-causes/syc-20369906 [accessed Aug 26, 2024]
17. Back Pain. National Library of Medicine; Available from: https://medlineplus.gov/backpain.html [accessed Aug 26, 2024]
18. Evaluation of low back pain in adults - UpToDate. Available from: https://www.uptodate.com/contents/evaluation-of-low-back-pain-in-adults?search=back%20pain&source=search_result&selectedTitle=1~150&usage_type=default&display_rank=1#H23 [accessed Aug 26, 2024]
19. Fainting. National Library of Medicine; Available from: https://medlineplus.gov/fainting.html [accessed Aug 26, 2024]
20. Low blood pressure (hypotension) - Symptoms and causes. Mayo Clin. Available from: https://www.mayoclinic.org/diseases-conditions/low-blood-pressure/symptoms-causes/syc-20355465 [accessed Aug 26, 2024]
21. Syncope in adults: Clinical manifestations and initial diagnostic evaluation - UpToDate. Available from: https://www.uptodate.com/contents/syncope-in-adults-clinical-manifestations-and-initial-diagnostic-

evaluation?search=syncope&source=search_result&selectedTitle=1~150&usage_type=default&display_rank=1#H50 [accessed Aug 26, 2024]
22. Vaginal Bleeding. National Library of Medicine; Available from: https://medlineplus.gov/vaginalbleeding.html [accessed Aug 26, 2024]
23. Heavy menstrual bleeding - Symptoms and causes. Mayo Clin. Available from: https://www.mayoclinic.org/diseases-conditions/menorrhagia/symptoms-causes/syc-20352829 [accessed Aug 26, 2024]
24. Common Cold. National Library of Medicine; Available from: https://medlineplus.gov/commoncold.html [accessed Aug 26, 2024]
25. Common cold - Symptoms and causes. Mayo Clin. Available from: https://www.mayoclinic.org/diseases-conditions/common-cold/symptoms-causes/syc-20351605 [accessed Aug 26, 2024]
26. General approach to drug poisoning in adults - UpToDate. Available from: https://www.uptodate.com/contents/general-approach-to-drug-poisoning-in-adults?search=overdose&source=search_result&selectedTitle=1~150&usage_type=default&display_rank=1#H19 [accessed Aug 26, 2024]
27. Opioid Overdose. National Library of Medicine; Available from: https://medlineplus.gov/opioidoverdose.html [accessed Aug 26, 2024]
